# Survey of activation‐induced genome architecture reveals a novel enhancer of *Myc*

**DOI:** 10.1111/imcb.12626

**Published:** 2023-02-14

**Authors:** Wing Fuk Chan, Hannah D Coughlan, Michelle Ruhle, Nadia Iannarella, Carolina Alvarado, Joanna R Groom, Christine R Keenan, Andrew J Kueh, Adam K Wheatley, Gordon K Smyth, Rhys S Allan, Timothy M Johanson

**Affiliations:** ^1^ The Walter and Eliza Hall Institute of Medical Research Parkville VIC Australia; ^2^ Department of Medical Biology The University of Melbourne Parkville VIC Australia; ^3^ Department of Microbiology and Immunology University of Melbourne at the Peter Doherty Institute for Infection and Immunity Melbourne VIC Australia; ^4^ School of Mathematics and Statistics The University of Melbourne Parkville VIC Australia

**Keywords:** B cell activation, B lymphocytes, chromosome conformation capture, differential enhancer usage, enhancer redundancy, Myc

## Abstract

The transcription factor Myc is critically important in driving cell proliferation, a function that is frequently dysregulated in cancer. To avoid this dysregulation Myc is tightly controlled by numerous layers of regulation. One such layer is the use of distal regulatory enhancers to drive *Myc* expression. Here, using chromosome conformation capture to examine B cells of the immune system in the first hours after their activation, we reveal a previously unidentified enhancer of *Myc*. The interactivity of this enhancer coincides with a dramatic, but discrete, spike in *Myc* expression 3 h post‐activation. However, genetic deletion of this region, has little impact on *Myc* expression, Myc protein level or *in vitro* and *in vivo* cell proliferation. Examination of the enhancer deleted regulatory landscape suggests that enhancer redundancy likely sustains *Myc* expression. This work highlights not only the importance of temporally examining enhancers, but also the complexity and dynamics of the regulation of critical genes such as *Myc*.

## Graphical abstract

In this article, we explored the three‐dimensional genome architectural changes in the genome of B cells in the hours after their activation. This revealed a previously undetected enhancer of the *Myc* gene. The interaction of this enhancer with the *Myc* promoter coincides with a dramatic, but discrete, spike in *Myc* expression 3 h post‐activation. However, genetic deletion of this region in mice, has little impact on *Myc* expression, Myc protein level or *in vitro* and *in vivo* cell proliferation, with enhancer redundancy likely sustaining Myc expression in the absence of the enhancer.

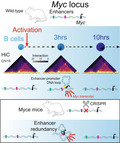

## INTRODUCTION

In order to mount an appropriate immune response, immune cells process activation signals from pathogens and immune accessory cells. The sum of these signals induces proliferation, differentiation and, in the case of lymphocytes, clonal expansion. The magnitude of this response is critical. An underestimation of the threat could be fatal, while an overestimation of the danger could lead to autoimmune damage. Thus, activation induces dramatic and rapid, but tightly controlled molecular changes in immune cells, including transcriptional,^1^ epigenetic,^2^, ^3^ proteomic,^4^ metabolomic^5^ and three‐dimensional genome organizational changes.^1^

Among the most archetypal and well studied of these changes are those that occur to the gene of the transcription factor *Myc* and its protein product.^6^ Upon lymphocyte activation, Myc is rapidly upregulated in a highly controlled manner in order to oversee further transcriptional changes central to immune cell activation.^7^, ^8^, ^9^

Unsurprisingly, given its importance in both the immune context and in all healthy and diseased cell proliferation,^10^ the body of research on the regulation of *Myc* is extremely rich.^11^, ^12^ Interestingly, many of the fundamental discoveries involving the regulation of *Myc* were made in immune cells. For example, shortly after the first reports of the existence and function of distal regulatory enhancers,^13^ *Myc* was among the first eukaryotic genes in which regulation by these gene regulatory elements was explored.^14^, ^15^ While these early studies explored the role of aberrant enhancement of *Myc* expression driven largely by translocation, the normal genomic location of *Myc*, within a large gene‐poor region in both mice and humans, made it an excellent model for the exploration of distal gene regulation. A large fraction of this regulation occurs in three‐dimensions, with distal enhancers being physically drawn to the *Myc* promoter to drive expression. Thus, it is unsurprising that the invention of chromosome conformation capture, which can reveal the three‐dimensional proximity of DNA to other DNA,^16^, ^17^ drove significant advancement of our understanding of the distal regulation of *Myc*.^11^ These studies revealed an elaborate, cell‐type‐ and cell‐state‐specific three‐dimensional enhancer landscape controlled by umpteen transcription factors, histone modifications, DNA methylation, long non‐coding RNAs,^18^ among other mechanisms.^11^

Here, *via* exploring changes in three‐dimensional genome organization in the first hours post‐B cell activation, we reveal a previously uncharacterized upstream enhancer of *Myc*, apparent for mere hours following activation, which accompanies the rapid and dramatic spike in post‐activation *Myc* expression. However, genetic removal of this enhancer leads to minimal impact on either *Myc* expression or Myc protein level, which is likely to be due to differential enhancer usage or enhancer redundancy sustaining critical levels of Myc.

## RESULTS

### Activation induces genome reorganization upstream of *Myc* promoter

**Figure 1:**
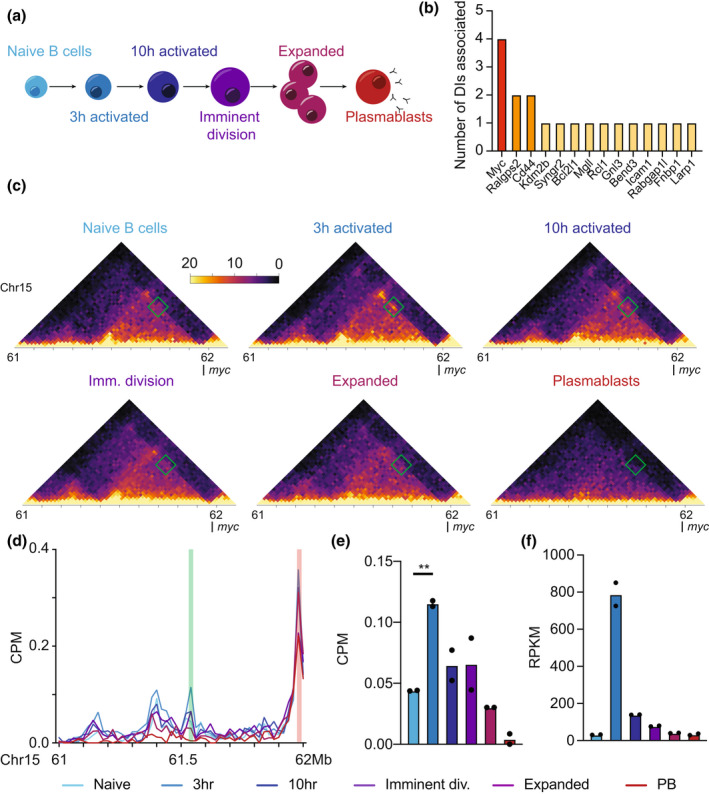
Activation‐induced genome reorganization upstream of *Myc* promoter. **(a)** Schematic of the stages of B cell differentiation examined using *in situ* HiC and RNA‐sequencing. **(b)** Number of differential interactions (DIs) associated with all DI‐associated differentially expressed genes (DEs) in the top 100 DEs between naïve and 3 h activated B cells. Libraries were generated from 3–12 mice. **(c)** Normalized *in situ* HiC contact matrices at 20 kbp resolution showing genomic region upstream of the *Myc* gene promoter of all six stages of B cell differentiation. The region shown is chr15:61–62.1 Mb. The color scale indicates the normalized counts per bin pair. The green box highlights the 3 h specific interacting region of interest. Libraries were generated from 3–12 mice. **(d)** Virtual 4C of the same region of chromosome 15 shown in **c**, at 20 kbp resolution from viewpoint of the *Myc* promoter (chr15:61983391–61990390) plotted as counts per million (CPM) in all six stages of B cell differentiation. The red highlight represents the viewpoint. The green highlight represents the 3 h specific interactive region of interest. The mean of duplicate biological replicate samples is shown. Libraries were generated from 3–12 mice. **(e)** CPM of interaction between the *Myc* promoter and the 3 h specific interactive region of interest across all stages of B cell differentiation. An unpaired two‐tailed *t*‐test was used to test for significance. **P < 0.005. Libraries were generated from 3–12 mice. **(f)** Reads per million per kilobase of *Myc* transcripts across all stages of B cell differentiation derived from RNA‐Sequencing. Libraries were generated from 3–12 mice.

To begin exploring the three‐dimensional genome architectural change that may regulate early activation‐induced transcriptional change, we examined paired chromosome conformation capture (*in situ* HiC) and RNA‐Seq data of activation‐induced B cell differentiation^1^ with a particular focus on the changes between naïve and 3 h activated B cells (Figure 1a).

First, we examined all the differential interactions (DIs) between naïve and 3 h activated B cells associated with the top 100 differentially expressed genes (DEs) between the same cells (Supplementary table 1). Differential interactions are statistically significant differences in the DNA–DNA interaction frequency between any two points on the linear genome between samples, determined using diffHiC,^19^ and denote changes in enhancer‐promoter DNA loops, or other genome architectural changes. The top 100 differentially expressed genes associate with just 19 DIs, four of which associate with the promoter of the *Myc* gene (Figure 1b). This result suggests that *Myc* is one of the few genes regulated by early activation‐induced genome architectural change in B cells.

To further define the nature and position of the *Myc* associated activation‐induced genome architectural changes, we generated contact matrices of the genomic regions containing the DIs (Figure 1c) and performed virtual 4C of the same region using the *Myc* promoter (2 kbp upstream and 5 kbp downstream) as the viewpoint (Figure 1d, viewpoint shown in red). This analysis revealed a previously unknown DNA–DNA interaction between the *Myc* promoter and an upstream region (Figure 1c, d in green). This statistically significant change in interactivity (*P* = 0.001, unpaired two‐tailed *t*‐test,) appears to be highly 3 h specific (Figure 1e) and is the strongest architectural change in the region (Supplementary figure 1) between naïve and 3 h activated B cells. Interestingly, the interactivity of this region and the *Myc* promoter (Figure 1e) across activation‐induced B cell differentiation is reflective of the expression pattern of *Myc* in the same cells (Figure 1f). This may reflect a regulatory role for the region in the expression of *Myc*.

### Deletion of one putative enhancer of *Myc* alters *Myc* expression

**Figure 2:**
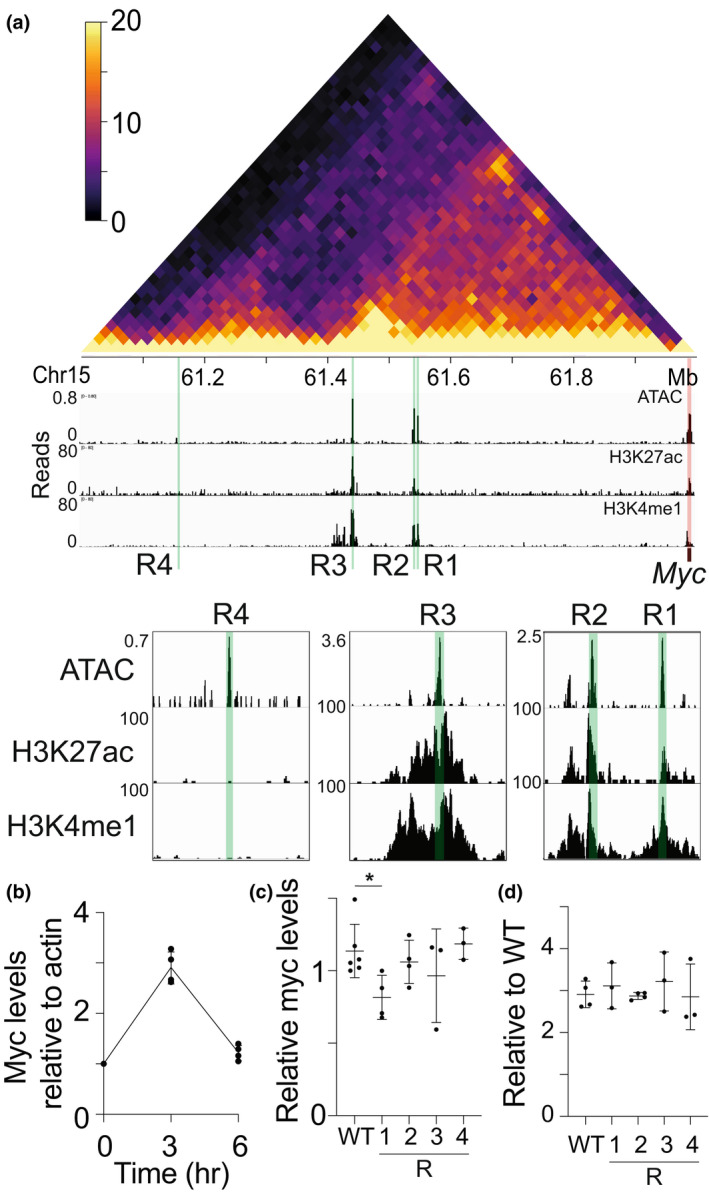
Genetic deletion of putative enhancer regions upstream of *Myc* reveals a novel enhancer. **(a)** Overlay of *in situ* HiC (top panel) from 3 h activated B cells at 20 kbp resolution and DNA accessibility (ATAC‐sequencing),^20^ H3K27 acetylation (H3K27ac)^21^ and H3K4 mono‐methylation (H3K4me1)^21^ from naïve B cells upstream of the *Myc* promoter (chr15:61‐62 Mb). Color scale indicates the normalized counts per bin pair. Regions 1–4 (R1‐4) are highlighted in green. The *Myc* promoter is highlighted in red. Insets show the same data at regions of interest at higher resolution. **(b)** Quantitative RT‐PCR of *Myc* transcript levels relative to *Actb* transcript (encoding β‐actin*)* in the A20 cell line 3 or 6 h post‐activation with lipopolysaccharide (LPS), relative to 0 h. Data are representative of three independent experiments and normalized to *Actb* at 0 h. Mean ± s.d. is shown. **(c)** Quantitative RT‐PCR of *Myc* transcript levels relative to *Actb* transcript in A20 cells with either region 1, 2, 3 or 4 (R1‐4) genetically deleted. Region 1–4 data are presented as relative to the wild‐type (WT) A20 sample. *P < 0.05. Data are representative of at least three independent experiments and normalized to *Actb*. Mean ± s.d. is shown. **(d)** Quantitative RT‐PCR of *Myc* transcript levels in A20 cells activated for 3 h with LPS with either region 1, 2, 3 or 4 (R1‐4) genetically deleted. Region 1–4 data are presented as relative to the levels prior to activation. Data are representative of at least three independent experiments and normalized to *Actb*. Mean ± s.d. is shown.

To explore potential gene regulatory roles for the putative activation‐induced enhancers discovered upstream of *Myc*, we first sought to clarify the potential functions of the regions using available epigenetic data. Overlaying our *in situ* HiC data with publicly available ATAC‐sequencing^20^ and H3K27 acetylation and H3K4 mono‐methylation chromatin immunoprecipitation data^21^ (generally associated with DNA accessibility, active enhancers and active/primed enhancers, respectively), we revealed part of the epigenetic landscape of the region in naïve B cells (Figure 2a). This analysis highlighted four genomic regions of particular interest (regions 1–4). Regions 1, 2 and 3 (R1‐3) were both accessible and contained epigenetic marks consistent with enhancers. Region 4 was accessible and in contact with the *Myc* promoter, but contained no detectable H3K27ac or H3K4me1 modifications.

**Figure 3:**
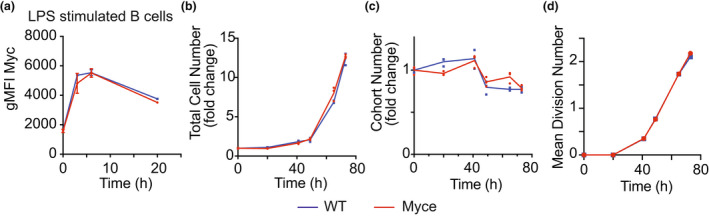
Myce mice have normal *in vitro* B cell activation responses. **(a)** Plot of geometric mean fluorescence intensity (gMFI) of Myc protein detected by flow cytometry in Myce mice or wild‐type littermate control B cells at 0, 3, 6 or 20 h post‐activation with LPS. **(b)** Fold change in total B cell number relative to 0 h across 72 h post‐activation with LPS in Myce mice and wild‐type littermate controls. **(c)** Cohort number (number of B cells in each division divided by 2 to the power of the division number) as a measure of B cell survival over 72 h post‐activation with LPS in Myce mice and wild‐type littermate control B cells. **(d)** Mean division number, measured by Cell Trace Violet division tracker, over 72 h post‐activation with LPS in Myce mice and wild‐type littermate control B cells. All data are representative of two independent experiments with 2 or 3 mice pooled per experiment. Mean ± s.e.m. is shown.

To definitively characterize the function of these regions in regulating *Myc* expression, we genetically removed each region using clustered regularly interspaced short palindromic repeats (CRISPR)/Cas9 technologies in the A20 B cell line (Supplementary figure 2a). First, we confirmed that activation induced *Myc* expression shows a similar pattern in the A20 cell line as in primary B cells (Figure 2b). We then quantified the impact of the genetic removal of regions 1, 2, 3 or 4 (Supplementary figure 2a) on *Myc* expression levels in the steady state (Figure 2c) or 3 h after activation (Figure 2d) using qRT‐PCR. Only deletion of region 1 in the steady state has any impact on the levels of *Myc*, inducing a small but significant reduction (Figure 2c). Interestingly, this reduction is no longer present in the same cells activated for 3 h (Figure 2d), suggesting different mechanisms function to regulate *Myc* expression after activation. To explore this putative activation‐induced regulatory program, we determined which transcription factor motifs were present in regions 1–4 and the 2 kb region upstream of the *Myc* transcriptional start site (promoter) with FIMO from the MEME suite. We filtered transcription factor motifs for only those differentially expressed between naïve B cells and 3 h activated B cells^1^ (calculated with fold changes significantly above 1.5 with TREAR and FDR < 0.05; Supplementary table 3) and normalized motif incidence to region size. We find no transcription factor motifs unique to R1; however, R1 does have the highest density of motifs of all regions examined (120 bp/motif) with a number of NFκb family members motifs represented, among others (Supplementary figure 2d). These transcription factors, particularly those that increase in expression post‐activation (Supplementary figure 2e), may influence the function of the enhancer or the formation of the enhancer‐promoter loop. Regardless of the mechanism, the impact on *Myc* expression upon genetic deletion of region 1 demonstrated that this region functions as an enhancer of *Myc*. To more fully explore its function, we generated a mouse strain with this region deleted (Supplementary figure 2b, c), termed Myce (*Myc* enhancer deleted) mice.

### Myce mice have normal *in vitro* B cell activation responses

After confirming that Myce mice have a normal immune compartment in various organs (Supplementary figure 3a–k) and normal antibody production (Supplementary figure 3l), we performed an *in vitro* B cell activation assay to quantify activation induced proliferation and survival. In brief, B cells were isolated from Myce mice or wild‐type littermate controls, activated with lipopolysaccharide (LPS) then their Myc protein levels, proliferation and death were observed over time. These experiments revealed little to no differences between Myce mice and littermate control B cells in either the levels of Myc protein in the early hours after activation (Figure 3a, Supplementary figure 3d), the total cell number in culture over their 3 day activation‐induced expansion (Figure 3b), or the B cells survival (Figure 3c) or division rate (Figure 3d) during this culture period. Similar results were seen in T cell activation cultures (Supplementary figure 3e–h). These experiments emphatically show that the deletion of the enhancer of *Myc* has no significant impact on either the activation‐induced levels of Myc, nor the B cell response to *in vitro* activation.

### Myce mice have normal *in vivo* B cell activation responses

**Figure 4:**
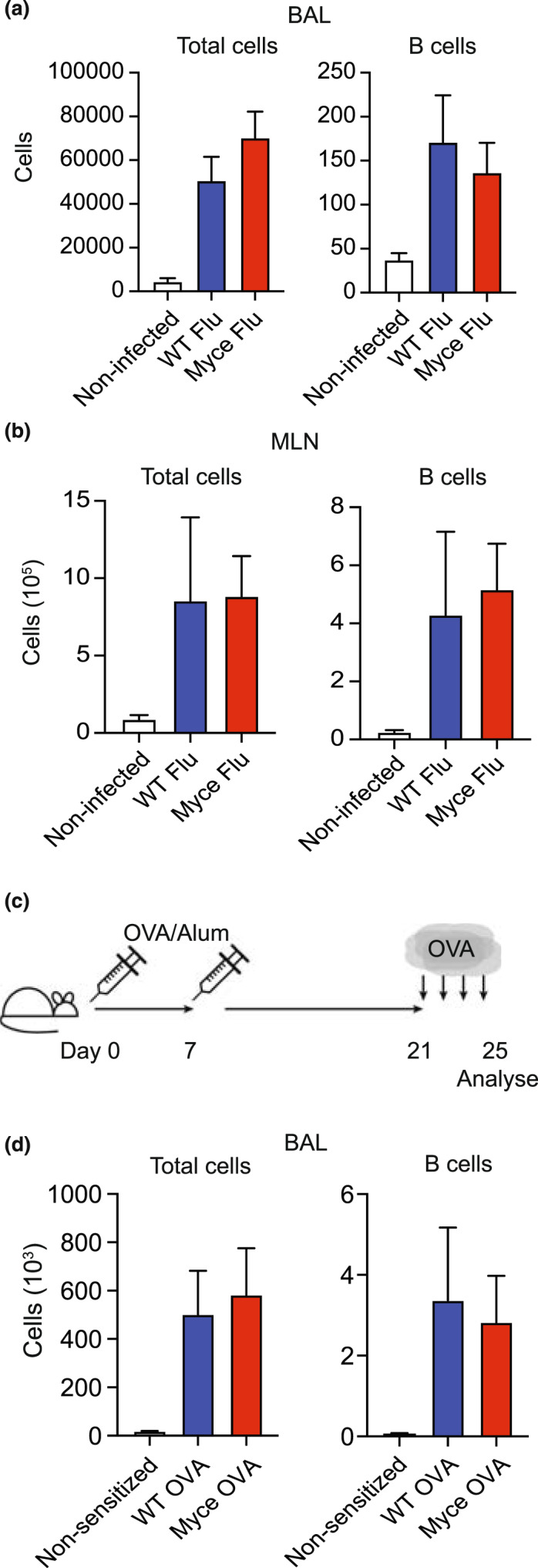
Myce mice have normal *in vivo* B cell activation responses. **(a)** Number of total live cells and B cells detected in the bronchoalveolar lavage (BAL) of 16 Myce mice, 16 littermate controls (WT Flu) or nine mice not infected with influenza (Non‐infected) at day 8 post‐infection. **(b)** Number of total live cells and B cells detected in the mediastinal lymph nodes (MLN) of six Myce mice, six littermate controls (WT Flu) or four mice not infected with influenza (Non‐infected) at day 8 post‐infection. **(c)** Schematic of the experimental setup for the model of asthma using OVA/alum sensitization. In brief, mice are sensitized with OVA and aluminum hydroxide on day 0 and 7, rested for 14 days then challenged with nebulized OVA for 4 consecutive days. Analysis is performed 1 day after the final OVA challenge. **(d)** Number of total live cells and B cells detected in the BAL of six Myce mice or six littermate controls (WT OVA) sensitized with OVA/Alum or two littermate controls sensitized with Alum alone (Non‐sensitized). Data are derived from at least two independent experiments. Mean ± s.e.m. is shown.

We next determined the impact of the deletion of the *Myc* enhancer on *in vivo* immune responses. First, we infected Myce mice and wild‐type littermate controls with influenza (X31). At the peak of the infection (day 8) we examined the total cell number and the B cell number in the bronchoalveolar lavage (BAL) (Figure 4a) and mediastinal lymph nodes (Figure 4b) of infected Myce mice and littermate controls (as well as uninfected littermate controls). This enumeration revealed no significant differences in lung infiltrate between the Myce mice and controls. Similarly, other immune cell types, both antigen specific and not, were not significantly affected by the enhancer deletion (Supplementary figure 4a, b).

We also tested the immune response generated in allergic lung inflammation using intraperitoneal ovalbumin sensitization followed by nebulized OVA challenge (Figure 4c).^22^ Cell populations in the BAL were examined the day after the final OVA challenge. Enumeration of total cells, B cells (Figure 4d) and other immune cell types (Supplementary figure 4c) revealed no significant differences between Myce mice and littermate controls.

Together with the unchanged numbers or proportions of immune cells in steady state Myce mice (Supplementary figure 3a–k), these experiments suggest that the deletion of this enhancer of *Myc* has little to no impact on the maintenance or activation response of immune cells *in vivo*.

### Myce mice cells display altered chromatin interactions with the *Myc* promoter

We next sought to determine why deletion of the *Myc* enhancer had little to no impact on either *Myc* expression, Myc protein levels or immune responses in cells from the Myce mice. To do so, we examined the consequence of the deletion on the genome architecture upstream of *Myc* in both the Myce mice and littermate controls. In brief, we isolated B cells from either Myce mice or wild‐type littermate controls, activated them *in vitro* for 3 h then sorted the activated and naïve B cells from the culture. A proximity ligation protocol followed by DNA precipitation was then performed on these cells, before qPCR was used to determine the frequency of DNA–DNA interaction between the *Myc* promoter and a number of upstream genomic regions.

**Figure 5:**
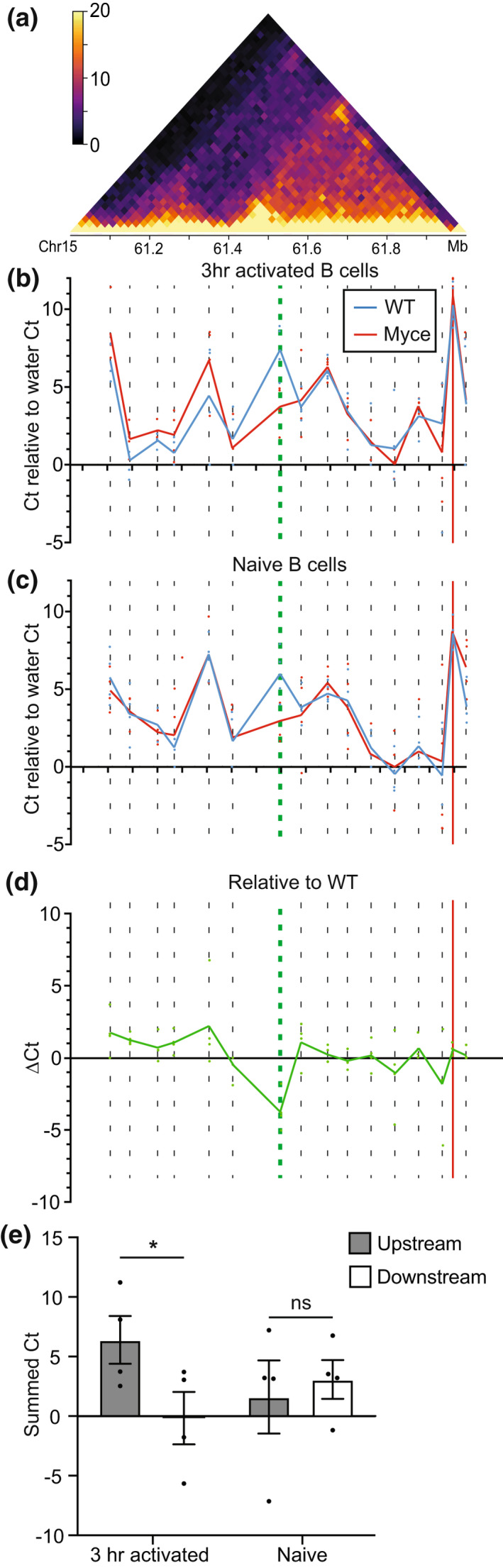
Altered interactions with the *Myc* promoter in Myce mice B cells. **(a)** *In situ* HiC contact matrix at 20 kbp resolution showing upstream of the *Myc* promoter (chr15:61‐62 Mb) in 3 h activated B cells. Color scale indicates the normalized counts per bin pair. **(b, c)** Threshold cycle (Ct) from qPCR targeting the *Myc* promoter and its proximity‐ligated DNA partners from across the region upstream of the promoter (Supplementary table 2), relative to a water control Ct, from **(b)** 3 h activated (CD69^+^) or **(c)** naïve (CD69^−^) Myce mice or wild‐type littermate control B cells. The dotted lines represent regions targeted for amplification by qPCR. The green dotted line represents the primer targeting the enhancer deleted region. The red vertical line represents the *Myc* promoter. Mean of four independent experiments is shown. **(d)** Threshold cycle (Ct) from qPCR targeting the *Myc* promoter and its proximity ligated DNA partners from across the region upstream of the promoter in activated Myce mice B cells relative to the activated wild‐type littermate control Ct. Dotted lines represent regions targeted for amplification by qPCR. The green dotted line represents the primer targeting the enhancer deleted region. The red vertical line represents the *Myc* promoter. The mean of four independent experiments is shown. **(e)** The sum of the Myce mice relative to littermate controls threshold cycle (Myce Ct ‐ WT Ct) from upstream and downstream of the deleted region (left and right of the green dotted line, respectively, in **d** in 3 h activated and naïve B cells. Mean ± s.e.m. from four independent experiments using a total of eight mice from each genotype is shown. *P < 0.05.

The frequency of interaction determined by this qPCR‐based method correlated well with *in situ* HiC data from the same region (Figure 5a–c) with regions of high or low interactivity detected similarly by both methods. Importantly, it also revealed that in 3 h activated B cells the interaction frequency at each examined region upstream of the deleted enhancer is greater in the Myce mice B cells than in littermate controls (Figure 5b, left of the green dotted line). This is in contrast to the regions downstream of the deletion in both Myce mice and littermate controls (Figure 5b, right of the green dotted line) and in naïve B cells either up‐ or downstream (Figure 5c), suggesting the change in interactivity is deletion and activation induced. This change in interactivity can be more clearly observed in the frequency of interaction in the activated Myce mice B cells relative to the wild‐type (Figure 5d). Furthermore, summing all relative threshold cycle (Ct) values upstream or downstream of the enhancer deleted region in both naïve and activated B cells reveals a significant (*P* = 0.04, paired two‐tailed *t*‐test) increase in interaction frequency upstream of the deletion, specifically in activated B cells (Figure 5e).

These experiments suggest that the reason that Myc levels, or indeed immune responses, are unchanged in the Myce mice B cells under various activation conditions is that in the absence of the enhancer the *Myc* promoter simply continues searching the upstream DNA to find another enhancer to sustain its expression. This is reflected in the increased interaction frequencies upstream of the enhancer deletion in Myce mice activated B cells.

## DISCUSSION

Here, through a comprehensive examination of genome organizational changes in the hours after B cell activation, we identified a previously undetected enhancer of *Myc*, in a highly scrutinized regulatory landscape.^11^ However, genetic deletion of this enhancer has little to no impact on *Myc* expression, protein levels or *in vitro* and *in vivo* cell proliferation or survival. Further exploration revealed that *Myc* expression is maintained in the absence of this activation‐induced enhancer likely *via* differential enhancer usage or enhancer redundancy.

Enhancer redundancy is the process by which gene expression is sustained upon the deletion of an enhancer of a gene by the action of other enhancers of the gene.^23^ This process has been widely documented across the kingdoms of complex life^24^, ^25^, ^26^, ^27^ and appears to be enriched in genes important in development and health, supporting robust transcription buffered against environmental and genetic perturbation. For example, the total number of predicted enhancers and the redundancy of transcription factor binding within these enhancers is predictive of a genes potential pathogenicity.^28^, ^29^ Experimentally testing this potential is challenging as the deletion of enhancers in these redundant regulatory landscapes frequently results in little to no phenotypic impact.^23^ However, this is not true in all cases. For example, in the *Myc* regulatory landscape, deletion of eight different enhancers within 2 megabases downstream of the *Myc* promoter,^30^, ^31^ or large sections of the region,^32^ each lead to significant reductions in *Myc* expression.

Our detailed exploration of the three‐dimensional organization in the enhancer deleted genome provides interesting insights into potential mechanisms of enhancer redundancy. As such, in 3 h activated B cells, deletion of the activation induced enhancer appears to increase DNA–DNA interactions between the *Myc* promoter and numerous upstream enhancers. Furthermore, the magnitude of these interaction changes suggests multiple new interactions per B cell. This suggests that the redundancy observed is not simply a case of the *Myc* promoter contacting and utilizing the next upstream enhancer to maintain appropriate regulation, but potentially the whole upstream regulatory landscape.

Why some enhancers exhibit redundancy and others do not is unknown. What is clear is that the combined function of enhancers is extremely context specific. For example, the enhancers of *hunchback* in *Drosophila melanogaster* will behave either additively (the induction of expression in the presence of both enhancers is the sum of both individually) or subadditively (the induction of expression in the presence of both enhancers is less than the sum of both individually) depending on the concentration of the transcription factor, Bicoid.^33^ Similarly, two enhancers of *pomc* in mice have been demonstrated to function additively in adult mice neurons, but superadditively (the induction of expression in the presence of both enhancers is greater than the sum of both individually) in young mice neurons.^34^ Thus, while it is clear that transcription factors can influence enhancer usage, epigenetics, the relative position of the enhancer to the promoter or other enhancers, among many other variables, likely impact enhancer redundancy. Transcription factors likely play a role in the enhancer redundancy observed in this study as the deleted enhancer was enriched for transcription factor motifs, including NFκb. NFκB has previously been shown to regulate *Myc* expression *via* binding near the promoter,^35^ so under normal conditions our enhancer likely forms part of this NFκB ‐*Myc* regulatory system.

Here we have revealed a slight, but significant, change in steady state *Myc* expression levels upon deletion of a novel enhancer, revealed by a detailed examination of genome organization just hours after cell activation. However, enhancer redundancy has made it challenging to accurately dissect the normal function of this enhancer. Nonetheless, the correlation between the temporal specificity of the interaction between our novel enhancer and the *Myc* promoter and the dramatic and discrete spike in *Myc* expression at 3 h post‐activation is compelling. Thus, we propose that under normal conditions this activation‐induced enhancer assists in regulating this critical and dramatic increase in *Myc* expression. The development and application of new super‐resolution live imaging technologies that allow concurrent visualization of multiple regions of DNA and associated factors at single molecule resolution,^36^, ^37^ will likely soon enable the visualization and dissection of not only this function, but the discussed genome architecture of the enhancer deleted *Myc* regulatory landscape and definition of the factors regulating redundancy.

## METHODS

### Generation of Myce mouse

For generating the Myce mouse strain, a 215‐bp region (chr15:61,418,510‐61 ,418 ,725) approximately 438 000 bp upstream of the *Myc* promoter on chromosome 15 was deleted by CRISPR editing using two single gRNAs with the sequences 5'‐ATAGATCATGACCTCCAAAG‐3′ and 5′‐ GACTTTGAACGTTACCTTCT‐3′ following the protocol by Aubery *et al*.^38^ Mice were genotyped using the forward primer 5'‐GTTTTATTTCTTCCCCAAGGCAC‐3′ and reverse primer 5'‐AGCACTGGGTCTCTGAACTG‐3′. A PCR product of 394 bp is expected if the deleted allele is present. Deletion was confirmed by gel electrophoresis and next generation sequencing (Supplementary figure 2b, c). Myce^−/−^ mice were generated on a C57BL/6J background and crossed to C57BL/6J for four generations prior to experimental use.

### Mouse immunophenotyping

Bone marrow of Myce and littermate wild‐type control were harvested into single‐cell suspension with red cell lysis and stained with CD19‐Pacific Blue (1D3, BD, NJ, USA), B220‐PE cy7 (RA3‐6B2, BD), CD24‐APC (M1/69, in house), IgM‐FITC (331.12, in house), CD43‐PE (S7, BD). Spleen red cell lysed single cell suspensions were stained with CD19‐BB700 (1D3, BD), B220‐APC (Thermo Fisher, Waltham, USA), CD23‐PE cy7 (B3B4, Thermo Fisher), CD21‐BV421 (7E9, BioLegend, San Diego, USA), CD138‐PE (281–2, BD). The peritoneal cavity wash was stained with CD19‐PE (ID2, in house), B220‐APC (RA3‐6B2, Thermo Fisher), CD23‐PE cy7 (B3B4, Thermo Fisher), CD5‐BB700 (53–7.3, BD), CD11b‐eFlour450 (M1/70, Thermo Fisher). Lymph node and thymus of single cell suspensions were stained with CD19‐BUV395 (1D3, BD), (TCRβ‐PE (H57‐597, BD), CD4‐BV421 (GK1.5, BioLegend) and CD8a‐PerCp/Cy5.5 (53–6.7, eBioscience, San Diego, USA). Flow cytometry analysis was performed on a BD LSRFortessa X‐20 analyzer (BD).

### *In vitro* cell culture and activation

B lymphocytes or the A20 cell line were cultured in RPMI 1640 with 2 mm GlutaMAX (Thermo Fisher), 50 μm β‐mercaptoethanol (Sigma‐Aldrich, Burlington, USA), and 10% heat‐inactivated fetal calf serum (FCS; Sigma‐Aldrich) and stimulated with 25 μg mL^−1^ lipopolysaccharide; Salmonella typhosa origin, Sigma‐Aldrich). CD8 T cells were stimulated using anti‐CD3 (10 μg mL^−1^ plate‐bound, clone 145‐2C11, WEHI antibody facility), anti‐CD28 (2 μg mL^−1^, clone 3751, WEHI antibody facility) and human IL‐2 (Peprotech, Rocky Hill, USA). CD8 T cell cultures contained 25 μg mL^−1^ anti‐mouse IL2 (clone S4B6, WEHI antibody facility), which neutralizes the activity of mouse but not human IL‐2 *in vitro*.^39^ For experiments in which the cell division number was tracked, the cells were labeled with 10 μm CellTrace Violet (CTV, Invitrogen, Waltham, USA), following the manufacturer's protocol.

### Intracellular Myc staining

For intracellular staining of Myc, the cells were harvested, centrifuged at 500× *g* at 4°C for 5 min before resuspension in fixation buffer (0.5% PFA (Sigma‐Aldrich) + 0.2% Tween 20 (Sigma‐Aldrich) in PBS) and incubation at 4°C for 24 h. The cells were washed with FACS buffer before pelleting at 500× *g* at 4°C for 5 min and maintained in FACS buffer at 4°C until staining of all samples within each experiment could be performed simultaneously. For staining, the cells were incubated with either anti‐Myc (clone D84C12, Cell Signaling, Danvers, USA) or a rabbit IgG isotype control antibody in PBS‐0.1% BSA (Sigma‐Aldrich) for 45 min at room temperature. The cells were washed with PBS‐0.1% BSA before incubating with anti‐rabbit IgG Alexa Fluor 647 (A21244, Life Technologies, Carlsbad, USA) in PBS‐0.1% BSA, for 45 min at room temperature. The cells were washed and flow cytometry analysis performed.

### Calculation of total cell and cohort number

The total cell numbers were calculated based on the ratio of live cells to counting beads detected by flow cytometry in each sample, after a known number of beads was added prior to analysis. Dead cell exclusion was performed using propidium iodide (0.2 μg mL^−1^, Sigma‐Aldrich). The cohort number and the mean division number over time were calculated based upon CellTrace Violet dilution, using the “precursor cohort method” as described previously.^40^

### Asthma induction and analysis

Allergic lung inflammation was induced in Myce and wildtype mice using intraperitoneal ovalbumin (OVA)/alum sensitization followed by nebulized OVA challenge as described previously.^22^ On the day after the final challenge, ~200 μL of blood was collected into non‐heparinized tubes by retro‐orbital bleeding, and the mice were then killed by CO_2_ inhalation after which BAL was performed (250 μL twice with sterile PBS). Flow cytometry was performed fresh on BAL cells using the following staining panel: CD19‐BUV395 Clone#1D3, CD8a‐PerCPeFluor710 Clone#52–6.7, SiglecF‐PE Clone#E50‐2440 (BD), Ly6c‐eFluor450 Clone#HK1.4, CD11c‐FITC Clone #N418, GR1‐PECy7 Clone#RB6‐8C5, TCRβ‐APCeFluor780 Clone #H57‐597 (eBioscience), CD4‐Alexa647 Clone#GK1.5, CD11b‐Alexa700 Clone#M1/70 from WEHI Antibody Facility (Supplementary table 4). Flow Cytometry was performed on a BD LSRFortessa X‐20 analyzer (BD). SYTOX Blue Dead Cell Stain was used to exclude dead cells from analysis and SPHERO Rainbow Beads (BD) were used to calculate absolute cell counts.

### Influenza infection and analysis

Myce^−/−^ and wildtype mice were infected with influenza A/H3N2/X31 virus at 1 × 10^4^ pfu in 25 μL by intranasal insufflation on day 0. On day 8, the mice were killed by CO_2_ inhalation after which BAL was performed (250 μL twice with sterile PBS). ~200 μL of blood was collected into non‐heparinized tubes by retro‐orbital bleeding, and the mice were then killed by CO_2_ inhalation after which BAL was performed (250 μL twice with sterile PBS). Flow cytometry was performed fresh on BAL cells using the following staining panel: CD19‐BUV395 Clone#1D3, CD8a‐PerCPeFluor710 Clone#52–6.7 (BD), CD11c‐FITC Clone #N418, GR1‐PECy7 Clone#RB6‐8C5, TCRβ‐APCeFluor780 Clone #H57‐597 (eBioscience), CD4‐Alexa647 Clone#GK1.5, CD11b‐Alexa700 Clone#M1/70 from WEHI Antibody Facility (Supplementary table 4). MHC Class I tetramers PA224‐BV421 and NP366‐PE (a generous gift from Professor Katherine Kedzierska, The University of Melbourne) were used to detect influenza‐specific CD8^+^ T cells. Flow cytometry was performed on a BD LSRFortessa X‐20 analyzer. SYTOX Blue Dead Cell Stain was used to exclude dead cells from analysis and SPHERO Rainbow Beads were used to calculate the absolute cell counts.

### Enzyme‐linked immunosorbent assay (ELISA *)*

Antibody binding to influenza hemagglutinin protein was measured by ELISA. The 96‐well Maxisorp plates (Thermo Fisher) were coated overnight at 4°C with 2 μg mL^−1^recombinant X31 HA ectodomain from A/Aichi/2/1968 (SinoBiological; 40 059‐V08H). The plates were blocked with 1% FCS in PBS before duplicate wells of serially diluted mouse plasma were added and incubated at room temperature for 2 h. Bound antibody was detected using 1:10 000 dilution of HRP‐conjugated anti‐mouse IgG (KPL) and the plates developed using TMB substrate (Sigma‐Aldrich), stopped using 0.12 m sulfuric acid and read at OD 450 nm. Endpoint titers were calculated using as the reciprocal serum dilution giving signal 2× background using a fitted curve (Graphpad Prism; 4 parameter log regression).

### *In vitro* transcription of sgRNA

The sgRNA used in this study were generated *via in vitro* transcription, as described previously.^41^ In brief, the transcription template was generated by PCR using Q5 high fidelity DNA polymerase (New England Biolabs, Ipswich, USA) with dNTP (Promega, Madison, USA) and an annealing temperature of 58°C. Universal primers as well as a specific primer bearing the sgRNA flanked by T7 promoter sequence and scaffold (as the format T7‐sgRNA‐scaffold) were used (Supplementary table 2). The PCR products were purified by DNA clean & concentrator‐25 (Zymo Research, Irvine, USA). *In vitro* transcription was performed by incubating 5 μg of transcription template with NTP, pyrophosphatase (Thermo Fisher), RNase inhibitor (Lucigen, Middleton, USA) and NxGen T7 RNA polymerase (Lucigen) at 37 °C for 18 h. The transcription template was then digested by TURBO DNase (Invitrogen, Waltham, USA) at 37 °C for 45 min. The remaining sgRNA was then purified by RNA clean & concentrator‐25 (Zymo Research).

### Ribonucleoprotein (RNP) assembly and delivery

For Cas9 RNP, 150 pmol of *in vitro* transcribed sgRNA was incubated with 100 pmol of recombinant Cas9 nuclease (Integrated DNA Technologies, Coralville, USA) at room temperature for 15 min. RNP with 100 pmol of electroporation enhancer (IDT) were subsequently transfected into cells *via* electroporation. Delivery into A20 cells was performed *via* 4D‐Nucleofector (Lonza, Basel, Switzerland) with buffer SF and pulse code FF113.

### Generating and confirming the deletion of putative *Myc* enhancers

Deletion of candidate regions in the A20 cell line genome was performed using electroporation delivered RNP containing paired sgRNAs (Supplementary table 2). At 48 h post‐electroporation, single cell clones were sorted into U‐bottom 96‐well plates and expanded as outlined above. A fraction of the expanded population was lysed in Direct PCR lysis reagent (Viagen, Cedar Park, USA) with proteinase K (Roche, Basel, Switzerland). Genotyping was performed directly from the lysates with region targeting primers (Supplementary table 2) and MyTaq Red Mix (Bioline, London, England) followed by gel electrophoresis to reveal biallelic knockout clones. Expected deletions are shown in Supplementary figure 2.

### Quantitative reverse transcription PCR (qRT‐PCR)

RNA was extracted using NucleoSpin RNA Plus (Macherey‐Nagel, Duren, Germany) with gDNA removal, 1 μg of RNA was then reverse transcribed with random hexamer and anchored oligo dT primers using SensiFast cDNA synthesis kit (Bioline). Quantitative PCR was performed on the diluted cDNA using SensiFast probe (Bioline) as per the manufacturers’ protocol with β‐actin acting as an endogenous reference. Primer and probe sequences are detailed in Supplementary table 2. Gene expression was normalized to the endogenous control and relative expression was evaluated using ΔΔCt method.

### *In situ* HiC

The *in situ* HiC data are available as GEO series GSE147467. The HiC libraries were processed and DIs were assessed as described previously.^1^

### 3C from HiC

Fixation, proximity ligation and DNA precipitation were performed as for *in situ* HiC,^42^ except the 0.4 mm biotinylated dATP (Life Technologies) in the end blunting step was replaced with 10 mm unbiotinylated dATP (New England Biolabs). The protocol was ceased after DNA precipitation and before DNA shearing. The proximity ligated DNA was then quantified using a Nanodrop (Thermo Fisher).

The frequency of proximity ligated fragments containing the *Myc* promoter and other downstream regions was then quantified on the BioRad C1000 Thermocycler using the SensiMix SYBR NO‐ROX kit (Bioline) with 60°C annealing temperature, water or 5 ng of proximity ligated DNA per 25 μL reaction in duplicate. Primers P1 and P4 (Supplementary table 2) were used to capture the ligation events between the *Myc* promoter itself. The P3 primer to all other regions numbered sequentially according to their location across the 1 Mb upstream of the *Myc* promoter (Supplementary table 2) were used to quantify interactions between the *Myc* promoter and other regions. Primer #22 targeted the Myce deleted region. Data were plotted as the average raw threshold cycle (Ct) in DNA samples per primer pair minus the average threshold cycle of the paired water controls. Relative to wild‐type (WT) was calculated by subtracting the Myce mice Ct value from the associated WT Ct.

### Virtual 4C analysis

The virtual 4C profiles were produced with the diffHic package v1.26.0 in R and then plotted using Graphpad PRISM. The *Myc* promoter was defined with the TxDb.Mmusculus.UCSC.mm10.knownGene package v3.10.0 and by applying the promoters function from the GenomicFeatures package v1.46.2 with upstream = 2 kbp and downstream = 5 kbp. Interactions between the *Myc* promoter and the entire genome were counted across all samples with the connectCounts function from diffHic using regions = *Myc* promoter with a filter set to 0 and the second.region = 20 kbp. The counts per million for each bin were calculated with the cpm function from the edgeR package v3.36.0.

### Visualization of HiC

Normalized contact matrices at a 20 kbp resolution were produced with the HOMER HiC pipeline for visualization. With the summed biological‐replicate tag directories, the analyzeHiC function was used with the ‐balance option. Contact matrices were plotted using the plotHic function from the Sushi R package v1.34.0.^43^ The color palette was inferno from the viridisLite package v0.4.0.^44^

### Motif analysis

The web based version of FIMO v5.5.0 (MEME suite) with default settings was used to scan for motifs matches in the sequences of the regions 1–4 and the 2 kb region upstream of the *Myc* transcriptional start site (promoter).^45^ We used the HOCOMOCOv11 core mouse mono motif database^46^ and motifs were matched to the transcription factor using annotation from https://hocomoco11.autosome.org/downloads_v11. Only motifs from transcription factors that are differential expressed between the 3 h activation B cells and resting B cells were used in the analysis. Heatmaps were plotted with pheatmap package in R.

### RNA‐sequencing analysis

The RNA‐seq data are available as GEO series GSE147496. The RNA‐Seq libraries were processed and differential expression was assessed as previously described.^1^

## AUTHOR CONTRIBUTIONS

**Wing Fuk Chan:** Conceptualization; formal analysis; investigation; methodology; project administration; validation; writing – original draft; writing – review and editing. **Hannah Coughlan:** Data curation; formal analysis; investigation; methodology; resources; software; writing – original draft; writing – review and editing. **Michelle Ruhle:** Formal analysis; investigation; writing – original draft. **Nadia Iannarella:** Investigation; methodology. **Carolina Alvarado:** Investigation; methodology. **Joanna R Groom:** Investigation; resources. **Christine Keenan:** Formal analysis; investigation; methodology. **Andrew J Kueh:** Methodology; resources. **Adam Wheatley:** Investigation; methodology. **Gordon Smyth:** Data curation; formal analysis; investigation; methodology; software. **Rhys Allan:** Conceptualization; funding acquisition; investigation; resources; supervision; writing – original draft; writing – review and editing. **Timothy Johanson:** Conceptualization; formal analysis; investigation; methodology; project administration; resources; supervision; writing – original draft; writing – review and editing.

## CONFLICT OF INTEREST

The authors declare no competing interests.

## CODE AVAILABILITY

DiffHic and edgeR, which are freely available from the Bioconductor repository (https://bioconductor.org/packages/release/bioc/html/diffHic.html and https://bioconductor.org/packages/release/bioc/html/edgeR.html, respectively) were used in this study. Versions used for this manuscript were: diffHic v1.26.0,^19^ edgeR package v3.36.0,^47^ Sushi R package v1.22.0,^43^ HOMER v4.11.^48^ Open access publishing facilitated by The University of Melbourne, as part of the Wiley ‐ The University of Melbourne agreement via the Council of Australian University Librarians.

## Supporting information

 

## Data Availability

*In situ* HiC data are available as GEO series GSE147467. The HiC libraries were processed and differential interactions were assessed as previously described. RNA‐seq data are available as GEO series GSE147496. The RNA‐Seq libraries were processed and differential expression was assessed as previously described.
