# Supplementary material for: Survey of activation‐induced genome architecture reveals a novel enhancer of Myc
Source: Immunol Cell Biol. 2023 Feb 14;101(4):345–57. doi: 10.1111/imcb.12626 (PMC10952581; doi:10.1111/imcb.12626)
Supplement: Supplementary file 1 [file IMCB-101-345-s001.docx]

**
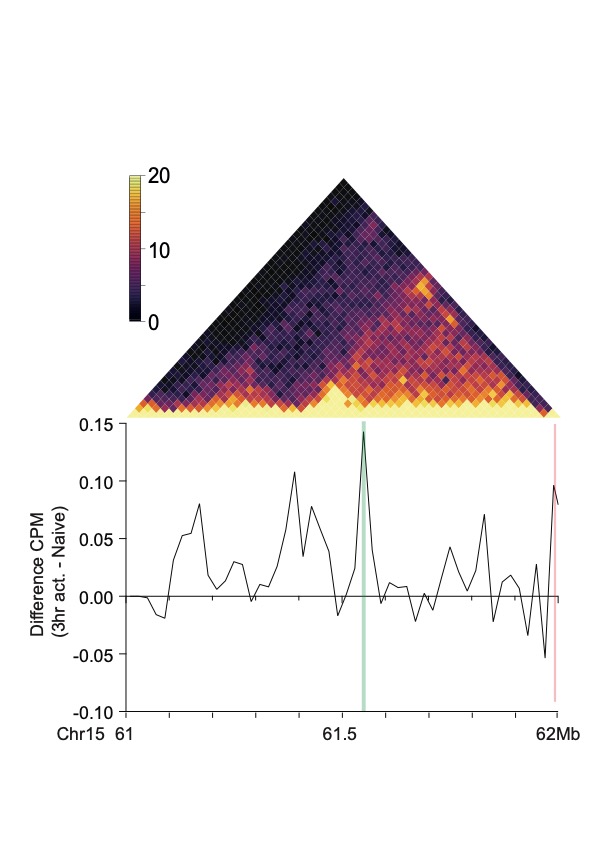
**

**Supplementary figure 1:** Difference between the pooled means of 3 hour activated B cell and naïve B cell *in situ* HiC libraries virtual 4C profiles (as plotted in Figure 1d) of region chr15:61-62Mb. Values are counts per million (CPM) in 20kbp bins relative to the viewpoint at the *Myc* promoter (chr15:61983391-61990390 bp). The vertical red line represents the viewpoint. The vertical green line represents the activation-induced enhancer. The libraries were generated from between 3-6 mice.


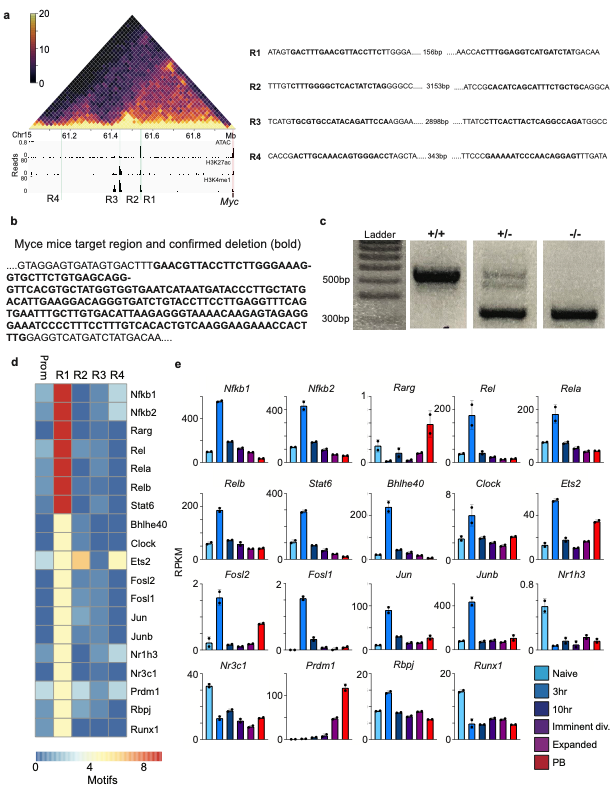


**Supplementary figure 2: (a)** The four genomic regions (R1-4) targeted for deletion using CRISPR/Cas9 in the A20 cell line, and sequences of the Cas9 target used to induce deletion (Supplementary table 2) **(b)** The genomic region (R1) deleted in the Myce mice line. Deletion was confirmed using Sanger and Next Generation sequencing. (**c)** Example of DNA band pattern derived from wild type, heterozygous and homozygous deletion of the enhancer of interest in Myce mice using PCR and gel electrophoresis. PCR primers are listed in Supplementary table 2. **(d)** Heatmap of density of transcription factor (differentially expressed between naïve and 3 hour activated B cells) motifs occurring in the R1 region (motif incidence per kbp) in the *myc* promoter (Prom) and regions 1-4 (R1-4) determined by FIMO. **(e)** Expression (RPKM) of transcription factors from (d) measured by RNA-Sequencing.

**
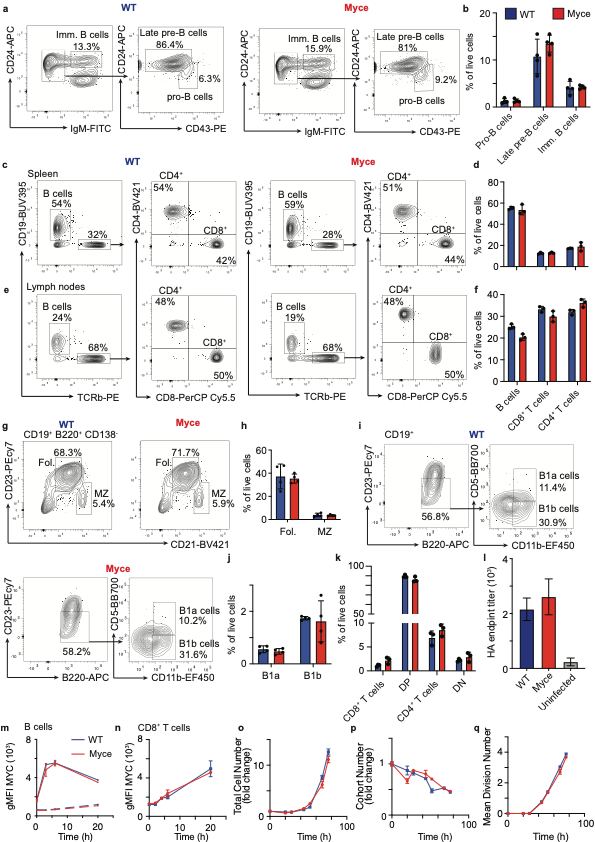
**

**Supplementary figure 3:** Flow cytometry profiles and quantitation of B cell progenitors in the bone marrow **(a, b)**, B and T cells in the spleen **(c, d)** and lymph nodes **(e,f)**, follicular and marginal zone B cells in the spleen (g), B1 cells in the peritoneal cavity **(i, j)** and T cells in the thymus **(k)** of Myce mice and littermate controls. Mean+/- SD shown. Data was generated from two independent experiments with 3-4 mice per genotype. **(l)** HA endpoint titre of antibodies against influenza haemagglutinin protein in the serum of Myce mice, wild-type littermate controls infected with influenza (A/H3N2/X31 virus at 1x10^4^pfu) or uninfected wild-type littermate controls measured by enzyme-linked immunosorbent assay. (**m)** The geometric mean fluorescence intensity (gMFI) of Myc staining in *in vitro* activated B cells derived from flow cytometry on Myce mice and wild-type littermate control B cells. Dotted lines indicate isotype control detection in the two populations. **(n)** Plot of geometric mean fluorescence intensity (gMFI) of Myc protein in Myce mice or wild-type littermate control CD8^+^ T cells at 0, 3, 6 or 20 hours post-activation with LPS. **(o)** Fold change in total CD8^+^ T cell number relative to 0 hours across 72 hours post-activation with LPS in Myce mice and wild-type littermate controls. **(p)** Cohort number (number of CD8^+^ T cells in each division divided by 2 to the power of the division number) as a measure of T cell survival over 72 hours post-activation with LPS in Myce mice and wild-type littermate control B cells. **(q)** Mean division number, measured by Cell Trace Violet division tracker, over 72 hours post-activation with LPS in Myce mice and wild-type littermate control CD8^+^ T cells. All data is representative of two independent experiments with 2-3 mice pooled per experiment. Mean +/- SEM shown.

** Supplementary figure 4:** Number of CD4^+^, CD8^+^ and tetramer positive T cells detected in the **(a)** bronchoalveolar lavage (BAL) or **(b)** mediastinal lymph nodes (MLN) of Myce mice and littermate controls infected with influenza (Influenza A/H3N2/X31at 1x10^4^pfu) and non-infected controls at Day 8 post-infection. 12 (BAL)-41 (MLN) total mice examined. **(c)** Number of total live cells and T cells detected in the bronchoalveolar lavage (BAL) of 6 Myce mice or 6 littermate controls (WT OVA) sensitised with OVA/Alum or 2 littermate controls sensitised with Alum alone (Non-sensitised). Data was derived from at least two independent experiments. Mean +/- SEM shown.

| **Supplementary table 1 Top 100 differentially expressed genes between naïve and 3 hour activated B cells and their associated differential interactions** | |
| --- | --- |
| Gene | # of DIs NBvs3HR |
| Myc | 4 |
| Ralgps2 | 2 |
| Cd44 | 2 |
| Kdm2b | 1 |
| Syngr2 | 1 |
| Bcl2l1 | 1 |
| Mgll | 1 |
| Rcl1 | 1 |
| Gnl3 | 1 |
| Bend3 | 1 |
| Icam1 | 1 |
| Rabgap1l | 1 |
| Fnbp1 | 1 |
| Larp1 | 1 |
| Nfkbiz | 0 |
| Il2ra | 0 |
| Traf1 | 0 |
| Siah2 | 0 |
| Zc3h12a | 0 |
| Srsf7 | 0 |
| Rhobtb2 | 0 |
| Wnt10b | 0 |
| Marcks | 0 |
| Srsf2 | 0 |
| Nfkb1 | 0 |
| Pogk | 0 |
| Cep250 | 0 |
| Epop | 0 |
| AI467606 | 0 |
| Wipf1 | 0 |
| Slc20a1 | 0 |
| Sytl3 | 0 |
| Nup62 | 0 |
| Ctps | 0 |
| Lbh | 0 |
| Tmem229b | 0 |
| Rab30 | 0 |
| Bcl2a1d | 0 |
| Ccnd2 | 0 |
| Rrs1 | 0 |
| Lyl1 | 0 |
| Marcksl1 | 0 |
| Adcy7 | 0 |
| Optn | 0 |
| Nfam1 | 0 |
| Gclc | 0 |
| Batf | 0 |
| Ndst1 | 0 |
| Tfrc | 0 |
| Slc25a19 | 0 |
| Sh2d3c | 0 |
| Ehd1 | 0 |
| Dck | 0 |
| Zmynd19 | 0 |
| Tgif1 | 0 |
| Nop58 | 0 |
| Ccdc86 | 0 |
| Ipcef1 | 0 |
| Ankrd33b | 0 |
| Sema7a | 0 |
| Insig1 | 0 |
| Bcl2a1b | 0 |
| Mrgpre | 0 |
| Nifk | 0 |
| Tnfrsf1b | 0 |
| Ppan | 0 |
| Dis3 | 0 |
| Hmgcr | 0 |
| Grwd1 | 0 |
| Jade2 | 0 |
| Ppat | 0 |
| Ceacam1 | 0 |
| Mphosph10 | 0 |
| Hhex | 0 |
| Nolc1 | 0 |
| Adm | 0 |
| Naf1 | 0 |
| Utp4 | 0 |
| Wdr75 | 0 |
| Slc39a6 | 0 |
| Plk3 | 0 |
| Mapk14 | 0 |
| Gadd45b | 0 |
| Calhm2 | 0 |
| Polr1b | 0 |
| Gm2a | 0 |
| Gpx1 | 0 |
| Klhl6 | 0 |
| Ggta1 | 0 |
| Rrp9 | 0 |
| Pus7 | 0 |
| Sptbn1 | 0 |
| Gng4 | 0 |
| Qtrt2 | 0 |
| Cd22 | 0 |
| Gnl2 | 0 |
| Elmsan1 | 0 |
| Rfc1 | 0 |
| Tnk2 | 0 |
| Swap70 | 0 |

| **Supplementary table 2 Primers used in the study** | |
| --- | --- |
| **Primer** | **Sequence** |
| **Region deletion in A20 cells** | |
| **Region 1 deletion** | GACTTTGAACGTTACCTTCT |
|  | ATAGATCATGACCTCCAAAG |
|  |  |
| **Region 2 deletion** | GCAGCAGAAATGCTGATGTG |
|  | CTTTGGGGCTCACTATCTAG |
|  |  |
| **Region 3 deletion** | TGCGTGCCATACAGATTCCA |
|  | CTTCACTTACTCAGGCCAGA |
|  |  |
| **Region 4 deletion** | AACTCCTGTTGGGATTTTTC |
|  | AGGTCCCACTGTTTGCAAGT |
|  |  |
| **Deletion in Myce mice** | ATAGATCATGACCTCCAAAG |
|  | GACTTTGAACGTTACCTTCT |
|  |  |
| **3C from HiC primers** |  |
| 1 | CTGTTGTCAGAAATTGCAAGCCCT |
| 3 | GGCTCAATGAGTTCTGAGACTGCT |
| 6 | GCAACCAGGGGCTCTCTTGATAGTA |
| 8 | GAAGCTTAGAGGGAGCTTGAATACATATGT |
| 12 | GGTGCTAGTCCAACTGGTGGT |
| 15 | GAGCACTGCCTTAAGTTAGACTGATTG |
| 22 | GTCACACTGTCAAGGAAGAAACCAC |
| 26 | CCTCAGTGGAAAGCCCTGAGTAC |
| 29 | CCCTCAGCTGGCTCTAGACTCA |
| 32 | CCTAGTCCACATGATTTCTGGAGC |
| 35 | GGCATTGCAGCCAAACTCTAAACTC |
| 38 | GCTGAGGTGCTGTTCTTGTGTG |
| 41 | GGAAGAATGCAGTGGACTCTCCTT |
| 44 | TGGTCCAGGGTTACATAACTGAGGTT |
| P3 | CTCTCGCTGGAATTACTACAGCGAG |
| P1 | AGGCGTCTCTCTAAGGCTGG |
| P4 | GTGGCAGTGAGTTGCTGAGCA |
| 50 | CATGGAGAGCCTTGACCTCCTT |
|  |  |
| **Myce genotyping primers** | |
| F - primary | CATAATGTGATTCTCAAGAGGAGAGTACATAC |
| F - deletion confirmation | GTCACACTGTCAAGGAAGAAACCAC |
|  |  |
| R | AGAAATGGAGCATGGGAGC |
| The R primer is used seperately with both the above F primers. Two PCRs are performed on each mouse to ensure geotyping accuracy. | |
|  |  |
| **Myc transcript qPCR (Taqman)** |  |
| Actb Forward | 5'-GACTCATCGTACTCCTGCTTG-3', |
| Actb Reverse | 5'-GATTACTGCTCTGGCTCCTAG-3', |
| Actb Probe | 5'-CTGGCCTCACTGTCCACCTTCC-3' |
| Myc Forward | 5'-CTTCCTCATCTTCTTGCTCTTCT-3' |
| Myc Reverse | 5'-TTCTCTCCTTCCTCGGACTC-3' |
| Myc Probe | 5'-CGGTGTCTCCTCATGCAGCACT-3' |
|  |  |
| **In vitro transcription of sgRNAs** | |
| Template sequence | AAAAAAAGCACCGACTCGGTGCCACTTTTTCAA-GTTGATAACGGACTAGCCTTATTTAAACTTGCTATG-CTGTTTCCAGCATAGCTCTTAAAC |
| Universal F | TATCCTAATACGACTCACTATAG |
| Universal R | AAAAAAAGCACCGACTCG |
| T7 promoter (append to 5' of sgRNA sequence) | ATCCTAATACGACTCACTATAG |
| Scaffold (append to 3' of sgRNA sequence) | GTTTAAGAGCTATGCTGG |
|  |  |
| **Putative enhancer deletion check primers** | |
| Myc R1 Forward | 5'-CATAATGTGATTCTCAAGAGGAGAGTACATAC-3', |
| Myc R1 Reverse | 5'-GTGCTCTTCCCAGGGAAAACTGTTTCC-3', |
| Myc R1 Internal | 5'-TGGGAGCAAGCAAACGATTTGC-3' |
| Myc R2 Forward | 5'-CTGTGGAACCTTGAGAAAGATGGGTTATAC-3' |
| Myc R2 Reverse | 5'-ATCCCTTTATGCAGTAGCCTGTATGTG-3' |
| Myc R2 Internal | 5'-GAGCAGGATGAAAGGCAAGGAC-3' |
| Myc R3 Forward | 5'-CTTGCTGCCCAAAATATTGTTTAGTGAAC-3' |
| Myc R3 Reverse | 5'-GAGGACTCACATTCCATCCAGATGAGAG-3' |
| Myc R3 Internal | 5'-CACTTGTGTGTAAGGGACATGTCATG-3' |
| Myc R4 Forward | 5'-TTTGTGAGGATTAACCACTGAAAGGTACG-3' |
| Myc R4 Reverse | 5'-CAATACCTGCTCACTGACAGCAAAC-3' |
| Myc R4 Internal | 5'-CTCTTTTGAAACAGCTTCTGCTTTGAAG-3' |

| **Supplementary table 3 Total number of motifs of transcription factors differentially expressed between naïve and 3 hour activated B cells detected in the *myc* promoter or regions 1-4.** | | | | | |
| --- | --- | --- | --- | --- | --- |
|  | Promoter | R1 | R2 | R3 | R4 |
| Nfkb1 | 3 | 2 | 0 | 2 | 1 |
| Nfkb2 | 2 | 2 | 0 | 2 | 1 |
| Rarg | 0 | 2 | 0 | 1 | 0 |
| Rel | 3 | 2 | 3 | 2 | 0 |
| Rela | 2 | 2 | 0 | 2 | 0 |
| Relb | 2 | 2 | 1 | 1 | 0 |
| Stat6 | 1 | 2 | 0 | 2 | 0 |
| Bhlhe40 | 0 | 1 | 2 | 0 | 0 |
| Clock | 0 | 1 | 2 | 0 | 0 |
| Ets2 | 5 | 1 | 21 | 1 | 2 |
| Fosl1 | 0 | 1 | 2 | 2 | 0 |
| Fosl2 | 0 | 1 | 3 | 2 | 0 |
| Jun | 0 | 1 | 4 | 2 | 0 |
| Junb | 0 | 1 | 2 | 2 | 0 |
| Nr1h3 | 2 | 1 | 0 | 3 | 1 |
| Nr3c1 | 0 | 1 | 0 | 1 | 0 |
| Prdm1 | 5 | 1 | 8 | 3 | 1 |
| Rbpj | 2 | 1 | 4 | 2 | 0 |
| Runx1 | 2 | 1 | 0 | 0 | 0 |
| Atf3 | 0 | 0 | 2 | 2 | 0 |
| Batf | 0 | 0 | 0 | 4 | 0 |
| Cbfb | 2 | 0 | 0 | 0 | 0 |
| Cebpa | 2 | 0 | 1 | 1 | 1 |
| Cebpb | 2 | 0 | 0 | 1 | 1 |
| Cebpe | 0 | 0 | 0 | 0 | 1 |
| Dbp | 1 | 0 | 3 | 0 | 0 |
| E2f6 | 4 | 0 | 3 | 0 | 0 |
| Elf1 | 0 | 0 | 4 | 0 | 1 |
| Epas1 | 1 | 0 | 0 | 0 | 0 |
| Ets1 | 1 | 0 | 7 | 0 | 1 |
| Fli1 | 3 | 0 | 6 | 0 | 1 |
| Foxj3 | 1 | 0 | 7 | 1 | 1 |
| Foxm1 | 0 | 0 | 2 | 2 | 0 |
| Foxo1 | 2 | 0 | 1 | 2 | 0 |
| Foxo3 | 2 | 0 | 0 | 2 | 0 |
| Gabpa | 0 | 0 | 5 | 0 | 0 |
| Gata2 | 0 | 0 | 1 | 1 | 0 |
| Gfi1b | 1 | 0 | 1 | 0 | 0 |
| Hic1 | 0 | 0 | 1 | 0 | 0 |
| Hnf1b | 1 | 0 | 0 | 2 | 0 |
| Hoxb4 | 0 | 0 | 1 | 0 | 0 |
| Ikzf1 | 1 | 0 | 1 | 1 | 1 |
| Irf1 | 3 | 0 | 9 | 8 | 0 |
| Irf2 | 3 | 0 | 31 | 6 | 0 |
| Irf4 | 3 | 0 | 8 | 2 | 1 |
| Irf8 | 5 | 0 | 8 | 3 | 1 |
| Irf9 | 1 | 0 | 1 | 2 | 0 |
| Klf1 | 8 | 0 | 7 | 3 | 0 |
| Klf6 | 11 | 0 | 11 | 4 | 0 |
| Lyl1 | 1 | 0 | 1 | 1 | 0 |
| Maf | 0 | 0 | 2 | 2 | 0 |
| Mafb | 0 | 0 | 3 | 1 | 0 |
| Maff | 2 | 0 | 2 | 2 | 1 |
| Mafg | 1 | 0 | 2 | 1 | 0 |
| Mafk | 2 | 0 | 2 | 2 | 0 |
| Mef2c | 0 | 0 | 1 | 2 | 1 |
| Mtf1 | 1 | 0 | 0 | 0 | 0 |
| Myc | 0 | 0 | 2 | 0 | 0 |
| Nfatc2 | 2 | 0 | 1 | 7 | 1 |
| Nfe2l1 | 0 | 0 | 2 | 0 | 0 |
| Nfil3 | 0 | 0 | 4 | 0 | 0 |
| Nr1d2 | 1 | 0 | 3 | 0 | 0 |
| Nr4a1 | 0 | 0 | 3 | 0 | 0 |
| Nr4a2 | 0 | 0 | 1 | 1 | 1 |
| Nrf1 | 1 | 0 | 0 | 0 | 0 |
| Ovol1 | 1 | 0 | 0 | 1 | 0 |
| Pax5 | 3 | 0 | 0 | 1 | 0 |
| Pbx1 | 0 | 0 | 1 | 0 | 0 |
| Pknox1 | 0 | 0 | 1 | 0 | 0 |
| Pou2f1 | 0 | 0 | 1 | 2 | 0 |
| Pou2f2 | 0 | 0 | 1 | 1 | 0 |
| Prdm9 | 1 | 0 | 0 | 0 | 1 |
| Rara | 0 | 0 | 1 | 1 | 0 |
| Rfx2 | 1 | 0 | 0 | 0 | 0 |
| Rfx3 | 0 | 0 | 0 | 2 | 0 |
| Runx2 | 2 | 0 | 2 | 1 | 0 |
| Runx3 | 1 | 0 | 0 | 0 | 0 |
| Smad3 | 0 | 0 | 4 | 0 | 1 |
| Smarca5 | 1 | 0 | 4 | 1 | 1 |
| Snai1 | 1 | 0 | 2 | 0 | 0 |
| Srebf2 | 6 | 0 | 2 | 1 | 0 |
| Stat1 | 23 | 0 | 76 | 7 | 2 |
| Stat2 | 14 | 0 | 11 | 6 | 0 |
| Stat3 | 1 | 0 | 0 | 2 | 0 |
| Stat4 | 1 | 0 | 1 | 1 | 1 |
| Stat5a | 1 | 0 | 2 | 4 | 0 |
| Tcf12 | 2 | 0 | 2 | 0 | 0 |
| Tcf7l1 | 1 | 0 | 0 | 1 | 1 |
| Tcf7l2 | 1 | 0 | 2 | 1 | 1 |
| Tead1 | 1 | 0 | 0 | 0 | 0 |
| Tfcp2l1 | 0 | 0 | 2 | 1 | 0 |
| Tgif1 | 0 | 0 | 0 | 1 | 0 |
| Thra | 2 | 0 | 2 | 1 | 0 |
| Usf1 | 0 | 0 | 4 | 0 | 0 |
| Zbtb17 | 26 | 0 | 25 | 2 | 0 |

| **Supplementary table 4 Antibodies used in this study** | | | | |
| --- | --- | --- | --- | --- |
| **Target** | **Fluorochrome** | **Clone** | **Source** | **RRID** |
| Anti-rabbit IgG | Alexa Fluor 647 |  | Life Technologies, A21244 | AB_2535812 |
| B220 | PEcy7 | RA3-6B2 | BD Biosciences | AB_394458 |
| B220 | APC | RA3-6B2 | Thermo Fisher Scientific | AB_469395 |
| CD11b | Alexa700 | M1/70 | In house |  |
| CD11b | eFluor450 | M1/70 | Thermo Fisher Scientific | AB_1582236 |
| CD11c | FITC | N418 | Thermo Fisher Scientific | AB_464940 |
| CD138 | PE | 281-2 | BD Biosciences | AB_395000 |
| CD19 | BUV395 | ID3 | BD Biosciences | AB_2722495 |
| CD19 | Pacific Blue | ID3 | BD Biosciences |  |
| CD19 | BB700 | ID3 | BD Biosciences | AB_2744310 |
| CD19 | PE | ID3 | In house |  |
| CD21 | BV421 | 7E9 | BioLegend | AB_10965544 |
| CD23 | PEcy7 | B3B4 | Thermo Fisher Scientific | AB_469604 |
| CD24 | APC | M1/69 | In house |  |
| CD4 | Alexa647 | GK1.5 | BioLegend | AB_493372 |
| CD43 | PE | S7 | BD Biosciences | AB_394748 |
| CD5 | BB700 | 53-7.3 | BD Biosciences | AB_2871364 |
| CD8a | PerCPeFluor710 | 52-6.7 | eBioscience | AB_1834433 |
| GR1 | PE cy7 | RB6-8C5 | Thermo Fisher Scientific | AB_469663 |
| IgM | FITC | 331.12 | In house |  |
| Ly6c | eFluor450 | HK1.4 | Thermo Fisher Scientific | AB_10805519 |
| Myc |  | D84C12 | Cell Signaling, #5605 |  |
| SiglecF | PE | E50-2440 | Thermo Fisher Scientific |  |
| TCRβ | APCeFluor780 | H57-597 | eBioscience | AB_1272173 |
